# Effects of mindfulness-based stress reduction on sleep quality and academic stress of Chinese adolescents: a randomized controlled trial

**DOI:** 10.3389/fpsyg.2026.1734353

**Published:** 2026-02-04

**Authors:** Jingxin Deng, Fang Xu

**Affiliations:** 1 School of Education Science, Hunan Normal University, Changsha, China; 2 College of Marxism, Hunan Normal University, Changsha, China

**Keywords:** academic stress, adolescents, controlled trial, mindfulness-based stress reduction, sleep quality

## Abstract

**Introduction:**

This study aimed to evaluate the impact of an 8-week Mindfulness-Based Stress Reduction (MBSR) program on sleep quality and academic stress among Chinese school adolescents.

**Methods:**

A total of 46 students were recruited and randomly assigned to either the experimental group (*n*=22), which participated in the 8-week MBSR program, or the control group (*n*=24), which received no intervention. Assessments using the Pittsburgh Sleep Quality Index (PSQI), the Academic Stress Questionnaire (ASQ), and the Five Factors Mindfulness Scale (FFMQ) were conducted before and after the intervention period.

**Results:**

Following the 8-week MBSR program, the experimental group demonstrated significant reductions in total scores for both sleep quality and academic stress, alongside a significant increase in total mindfulness score. In contrast, the control group showed no significant changes in academic stress, sleep quality, or mindfulness scores over the same period.

**Discussion:**

The findings indicate that the 8-week MBSR program effectively improved sleep quality, reduced academic stress, and enhanced mindfulness levels in Chinese adolescents, suggesting its potential as a beneficial intervention in school settings.

## Introduction

Adolescence is the critical developmental period for the rapid development of individual physiology and psychology. More and more attention has been paid to the psychological and behavioral problems faced by adolescents, such as academic stress can significantly predict the sleep quality (Schonert-Reichel and Lawlor, 2010). Sleep quality is to describe an individual’s sleep condition from both qualitative and quantitative perspectives (Buysse et al., 1989). It is worth noting that in a meta-analysis of 21 studies conducted in 2023, researchers found that the detection rate of sleep problems among Chinese junior high school students was as high as17% (Zhou et al., 2023). Poor sleep quality not only leads to physical health problems, psychological problems, interpersonal relationship problems, etc. among teenagers (Jin, 2023), but also affects their academic performance (Wang et al., 2022). Poor sleep quality also have a negative impact on adolescents’ social adjustment, causing negative emotional experiences such as anxiety and depression (Danielsson et al., 2013), and damaging executive functions including working memory and inhibitory control (Wang and Wang, 2015). Academic stress is a bad experience caused by students’ feelings of frustration when they are almost incompetent for learning tasks (Chen, 2004). Excessive academic stress can also lead to a series of emotional and cognitive problems, and even lead to poor academic performance, sleep disorders and other serious psychological and behavioral problems (Andrew et al., 2015; Teris and Paul, 2015). Therefore, it is imperative to explore effective intervention methods to improve sleep quality and academic stress. Such interventions can help adolescents better cope with the negative effects of stress, improve psychological problems, and improve the quality of life and learning.

Mindfulness-based stress reduction (MBSR) was originally developed by Kabat-Zinn and his colleagues at the University of Massachusetts Stress Reduction Clinic, and was initially used to treat chronic pain and stress, but has since been popularized to treat a variety of mental health problem (Kabat-Zinn, 1982). The MBSR program follows a structured curriculum that is taught in a group format (no more than 40 people) with 8 weeks (Kabat-Zinn, 2003). During the treatment, the therapist develops a training program based on the specific situation and applies mindfulness techniques such as breathing exercises, sitting meditation and body scanning. The therapy emphasizes the need for participants to focus on the present, reduce negative self-evaluation, become more aware of their physical and mental experiences, and ultimately achieve peace of mind. Mindfulness is the theoretical basis of clinical intervention of MBSR program. The three axioms of mindfulness of Shapiro et al. (2006) can explain how mindfulness interventions work. They hypothesized that mindfulness involves three factors: intention, attention and attitude. Intention is a central component of mindfulness training and reveals the purpose of mindfulness practice - self-regulation and stress management. Attention is the “return to itself,” the ability to pay attention to (observe) internal and external behavior. At the heart of mindfulness training is the practice of paying attention. Attitude is the quality of attention, emphasizing the absence of evaluation and interpretation while focusing on internal and external experiences. That is to say, MBSR training requires participants to shift their attention to the present moment and the event itself, not to the past (rumination) and future (anxiety), and to keep calm. They are interwoven aspects of a single cyclic process and occur simultaneously. Mindfulness is this moment-to-moment process.

Research has predominantly focused on the effects of mindfulness training on clinical populations, such as individuals with cancer recurrence (Mei Huiting et al., 2025), chronic pain (Jiating et al., 2024), and mind-wandering (Shao et al., 2023). In addition, researchers have applied mindfulness therapy to psychological domains such as stress and emotion. Previous studies have shown that mindfulness is significantly effective in improving stress, but its intervention effects on emotion are generally moderate (Yu et al., 2019). Previous studies have showed that MBSR program is beneficial in improving stress and sleep quality. Mindfulness can negatively predict perceived stress (Rodriguez et al., 2015), indicating that the higher the level of mindfulness, the lower the level of perceived stress. Compared to individuals with low perceived stress, those with high perceived stress are more prone to experiencing negative emotions such as anxiety and depression (Bergdahl and Bergdahl, 2002). Consequently, they are at a higher risk of developing psychological issues like anxiety and depression, or even suffering from depressive disorders. Mindfulness intervention has effective and significant effects on improving stress levels (Taylor et al., 2020) and stress coping ability (Smith et al., 2019). According to the research of Donald et al. (2016), the awareness of the present in mindfulness helps individuals cope with the stress in daily life. According to the stress buffer theory of Cohen and Wills (1985), mindfulness can act as a buffer against stress and reduce negative evaluations of stress responses (Creswell and Lindsay, 2014). Meanwhile, it has been reported that mindfulness intervention has a significant effect on reducing sleep disorders and improving sleep quality in patients with chronic diseases (Carlson and Garland, 2005), and this effect remains good in follow-up tests after 3 and 6 months (Ong et al., 2014). Bootzin and Stevens (2005) found improvements in sleep quality, mood, and mental health after 6 weeks of mindfulness stress reduction training in middle school students aged 13 to 19. In Bei et al. (2013) ‘s study, 10 students with poor sleep were selected from 62 middle school students (aged between 13 and 15) to conduct Mindfulness Stress Reduction (MBSR) intervention. Results showed that participants’ sleep time was prolonged, sleep efficiency was improved and overall sleep quality was improved after intervention. People who are prone to insomnia tend to worry excessively about their sleep and the consequences of poor sleep. When sleep problems occur, people with insomnia often think repeatedly about how to fall asleep, which will make it more difficult for them to fall asleep, resulting in sleep disorders and triggering automatic arousal (Harvey, 2002).

The purpose of the current study is to examine the feasibility and effectiveness of an adapted version of MBSR on sleep quality and academic stress of Chinese adolescents. Based on prior research documenting the effectiveness of MBSR in adults and some students (see above), we anticipated that Chinese adolescents who completed the MBSR program would demonstrate significant improvements in sleep quality and academic stress, especially in the context of China’s examination system.

## Method

### Participants

**Figure 1:**
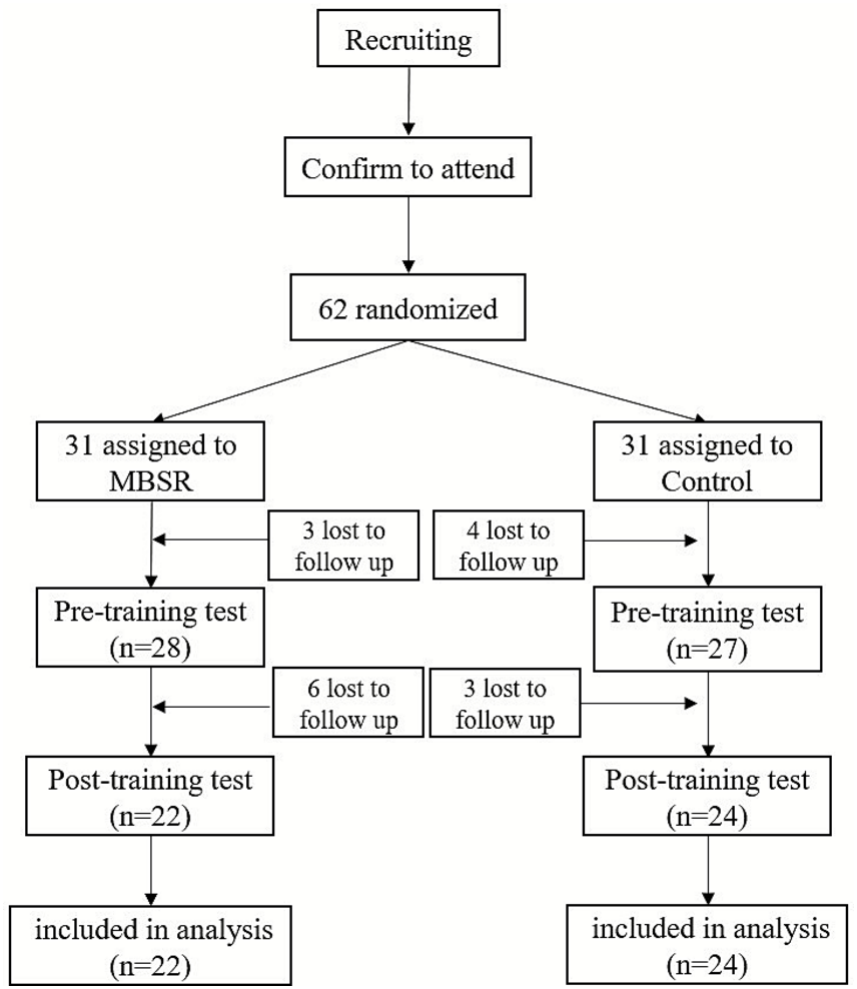
Flowchart of the control experiment.

A total of 62 students signed up for MBSR program at a middle school in Changsha, Hunan province. We randomly assigned these students to an experimental group of 31 (15 boys) and a control group of 31 (16 boys). During the subsequent test and training, a total of 15 participants dropped out including 8 girls and 7 boys (see Figure 1). One of them withdrew due to sudden illness; 1 person quit due to the objection of his guardian; 3 people quit due to time conflict in school activities; 5 people did not participate in the test due to leave; 5 dropped out because they lost interest in the experiment. In the end, a total of 46 subjects completed the experiment, including 22 in the experimental group (10 boys) and 24 in the control group (12 boys). The guardians and class teachers of all the participants were informed and agreed, and the guardians signed the informed consent for the study. The study was approved by the Hunan Normal University Ethics Committee (NSK-2020320).

### Measures

#### Academic stress

Academic stress was measured by the Academic Stress Questionnaire (ASQ) (Chung, 2018). It consists of five stress factors: (1) target requirement, (2) competition, (3) frustration, (4) expectation, and (5) self-development. It includes 56 self-rated items. Each item is scored from 1 (full disagreement) to 5 (full agreement). The questionnaire has satisfactory reliability: internal consistency reliability = 0 0.90; split-half reliability = 0.91; test–retest after 3 weeks reliability = 0.96 (Chung, 2018). In this study, the internal consistency of the full questionnaire (Cronbach′s *α*) was 0 0.97.

#### Sleep quality

Sleep quality over the past month was assessed with the Chinese version of Pittsburgh Sleep Quality Index (PSQI), which was based on Buysse et al. (1989). The Chinese version of the PSQI have good validity and internal consistency, Cronbach’s *α* = 0.75–0.85 (Lu et al., 2014), and test–retest reliability (0.85 over 14- and 21-day intervals) (Tsai et al., 2005). The seven subscale scores (subjective sleep quality, sleep latency, sleep duration, habitual sleep efficiency, sleep disturbances, use of sleep medication, and daytime dysfunction) were summed to calculate a global score. Scores on the PSQI ranged from 0 to 21, with higher scores indicating poorer sleep quality. People with a PSQI score greater than seven were defined as “poor sleepers,” Cronbach’s *α* was 0.73 in this study.

#### Mindfulness

The Five Facet Mindfulness Questionnaire (FFMQ) prepared by Baer et al. (2006) was used to measure Mindfulness. This study adopts the Chinese version revised by Deng et al. (2011), with 39 questions in total. The scale is divided into five dimensions: observe, describe, act with Awareness, non-judge, and non-react. The higher the total score, the higher the level of mindfulness. In this study, the Cronbach’s α of the scale was 0.79.

### Procedure

A quasi-experimental pretest–posttest comparison group design was utilized to assess the effectiveness of the adapted MBSR program. A convenience sample of 62 school students drawn from mental health class were recruited to participate in the study. Then the subjects who volunteered to participate in the training were interviewed. You cannot participate in this training if you have the following conditions. Students with chronic conditions such as sleep and stress; Students with mental disorders or other physical diseases; Students who have undergone mindfulness training within one year. Then they were randomly divided into experimental group and control group. Sleep quality and mindfulness scales were measured in both the intervention and control groups at the same time and place. After the pre-test, the experimental group was given 8 weeks of MBSR training, while the control group was given no training.

Students in the experimental group participated in an 8-week MBSR program delivered by a professional MBSR instructor. The experimental group participated in a 90-min mindfulness-based stress reduction program every Tuesday afternoon for eight weeks. During eight weeks of training, the experimental group was taught a variety of mindfulness techniques, including mindful breathing, mindful meditation, body scan, and mindful yoga. The program consists of theoretical teaching, practical exercises and group discussion. At the end of the weekly mindfulness training, the experimenter would assign homework to the participants in the experimental group. Participants were required to practice for 30–45 min every day and to fill in a mindfulness diary to record the practice process and questions. The mindfulness diary documents a set of fixed items, including practice content, duration, bodily sensations during practice, as well as emotions, thoughts, and attentional states. After eight weeks, sleep quality, academic stress, and mindfulness scales were measured in both the intervention and control groups. Finally, the collected data were analyzed and the subjective report materials of the experimental group were collated. To ensure data accuracy, all data entries were double-checked by two independent researchers. The two datasets were then compared electronically/in a blinded manner. Any inconsistencies between the entries were adjudicated and corrected by referring to the original raw data. The purpose of this training is to enhance participants’ cognition of their own behavior and thinking, exercise effective control of their attention, and form a receptive and open attitude.

### Analyses

In this study, SPSS 23.0 was used for data collation and analysis. The descriptive statistics were used to generalize the demographic data, and the independent sample t-test and the paired sample t-test were used for inter-group and intra-group comparisons of the variables.

## Results

### Demographics

48 students participated in this study, 21 girls and 25 boys. The age of participants ranged from 12 to 15 years old. The study found no gender or grades differences in sleep quality. See Table 1 for details.

**Table 1:** Demographic characteristics.

Group	*N*	Age (*M* ± *SD*)	Sex		Grade		
			Boy	Girl	7th	8th	9th
Experimental group	22	13.25 ± 2.33	11	11	6	9	7
Control group	24	13.58 ± 2.47	14	10	8	10	6

This table presents the demographic characteristics of the experimental and control groups, including sample size (N), age (M ± SD), gender distribution, and grade level distribution.

### The preliminary analysis

The preliminary analysis found that 50% of the sample in the experimental group met the clinical cutoff of 5 or higher for the PSQI score before beginning the intervention. More significantly, 11.0% of the sample had PSQI scores of 10 and greater. Comparatively, after completing the MBSR program, only 18.1% of the sample was still reporting PSQI scores above the score of 5 or greater (Table 2).

**Table 2:** PSQI scores pre and post MBSR intervention.

Category	Over 5	Over 10
Pre	11(50.0%)	2(11.0%)
Post	4(18.1%)	0

The data presented show the number and percentage of participants with PSQI total scores 5 (indicating poor sleep quality) and 10 (indicating severely impaired sleep quality).

The scores of the sleep quality subscale in the experimental group were decreased, in which the scores of subjective sleep quality, sleep latency, sleep duration, habitual sleep efficiency, sleep disorders, sleep medication use, and daytime dysfunction were all decreased. At the same time, the subscale of academic stress also improved, after eight weeks of MBSR, the competition, target requirement, frustration, expectation score of the experimental group were reduced.

### Effectiveness test of MBSR

The mean values and standard deviations of sleep quality, academic stress, meditation and scores of each dimension of the experimental group and the control group are shown in Table 3. Using independent sample t test analysis of baseline data experimental group and control group, according to the results, experimental group and control group before intervention sleep quality, academic stress and mindfulness scores had no significant difference (*p* 0.05), different variables of the experimental group and control group before intervention is homogeneous, grouping is also reasonable.

**Table 3:** Means and standard deviations of experimental and control groups at baseline and post intervention.

Variables		Experimental group		Control group	
		Baseline	Post	Baseline	Post
Sleep quality		7.27 ± 1.69	4.45 ± 1.41	6.88 ± 2.15	6.75 ± 1.62
	Subjective sleep quality	1.32 ± 0.64	0.73 ± 0.70	1.33 ± 0.72	1.29 ± 0.62
	Sleep latency	1.50 ± 0.67	0.86 ± 0.64	1.29 ± 0.75	1.17 ± 0.91
	Sleep duration	1.41 ± 0.50	0.86 ± 0.56	0.96 ± 0.75	1.17 ± 0.70
	Habitual sleep efficiency	0.55 ± 0.59	0.32 ± 0.47	0.42 ± 0.50	0.42 ± 0.50
	Sleep disturbance	1.05 ± 0.48	0.82 ± 0.39	1.21 ± 0.58	0.83 ± 0.63
	Use of sleep medication	0.18 ± 0.39	0.00 ± 0.00	0.08 ± 0.28	0.08 ± 0.28
	Daytime dysfunction	1.27 ± 0.73	0.86 ± 0.71	1.32 ± 0.64	1.32 ± 0.64
Academic stress		125.18 ± 25.33	95.45 ± 5.07	123.21 ± 20.05	121.83 ± 26.62
	Competition	21.41 ± 5.77	14.91 ± 2.41	23.79 ± 5.90	20.67 ± 5.24
	Target requirement	36.68 ± 8.19	28.18 ± 3.05	31.38 ± 7.62	36.46 ± 8.49
	Frustration	24.68 ± 5.66	19.77 ± 3.29	29.08 ± 7.73	26.67 ± 7.35
	Expectation	36.73 ± 9.22	27.45 ± 3.37	32.00 ± 5.03	31.38 ± 7.17
	Self-development	5.68 ± 2.36	5.14 ± 1.85	6.96 ± 2.14	6.67 ± 2.87
Mindfulness		115.73 ± 15.50	133.73 ± 10.46	110.75 ± 12.74	108.25 ± 7.37
	Observe	20.55 ± 7.37	30.23 ± 4.98	19.42 ± 6.95	20.33 ± 5.19
	Describe	23.41 ± 6.05	31.27 ± 3.05	23.42 ± 5.38	22.21 ± 4.43
	Act with Awareness	31.23 ± 6.32	28.18 ± 3.43	28.17 ± 7.79	26.42 ± 5.86
	Non-Judge	24.73 ± 5.34	17.73 ± 5.28	23.04 ± 5.12	20.63 ± 5.01
	Non-React	15.82 ± 4.72	26.32 ± 3.58	16.71 ± 4.99	18.67 ± 4.65

This table presents the means and standard deviations (M ± SD) of scores on sleep quality, academic stress, and mindfulness variables at baseline (pre intervention) and post intervention for the experimental and control groups.

**Figure 2:**
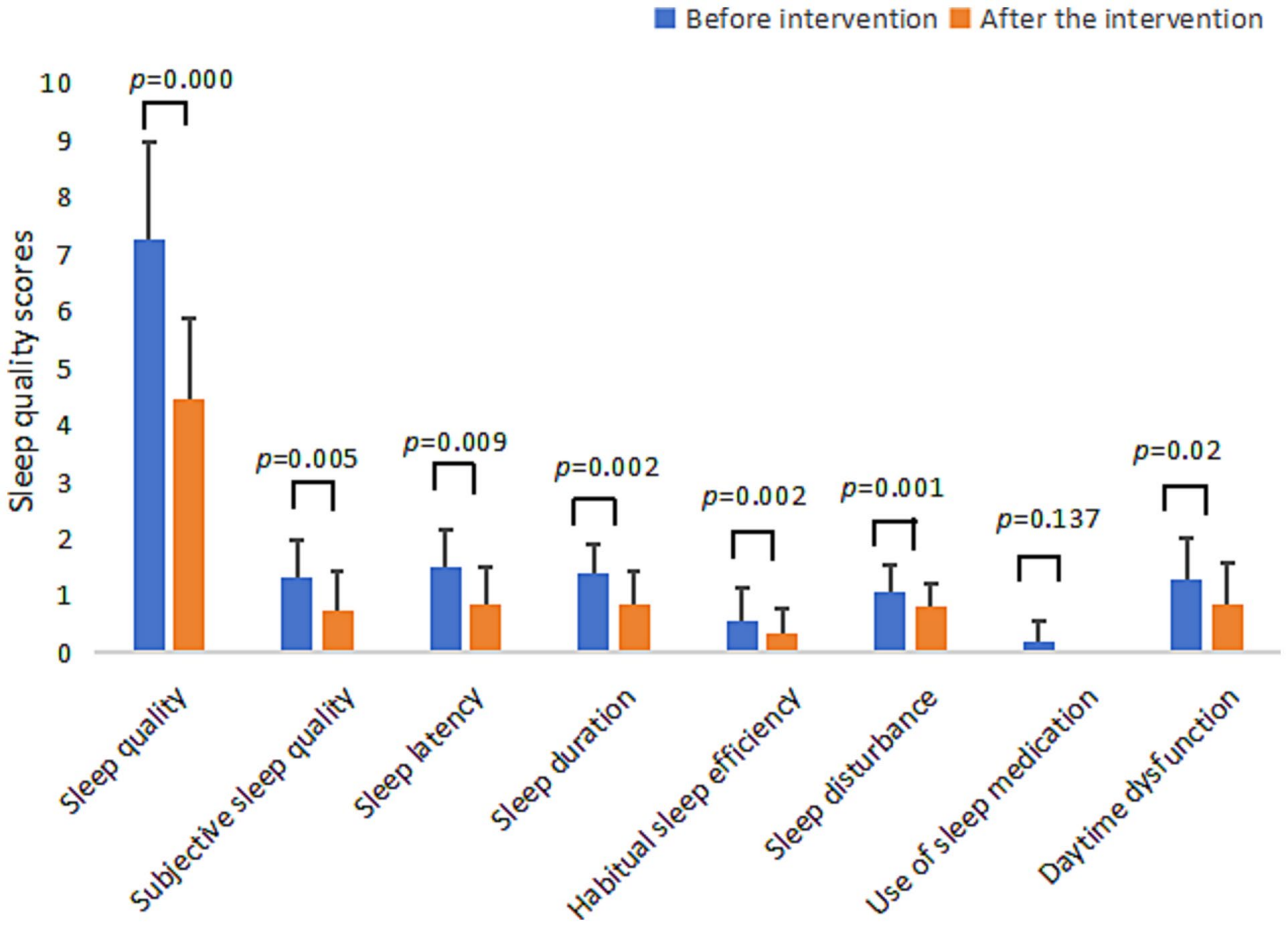
Bar chart of changes in sleep quality scores of the experimental group before and after the intervention.

**Figure 3:**
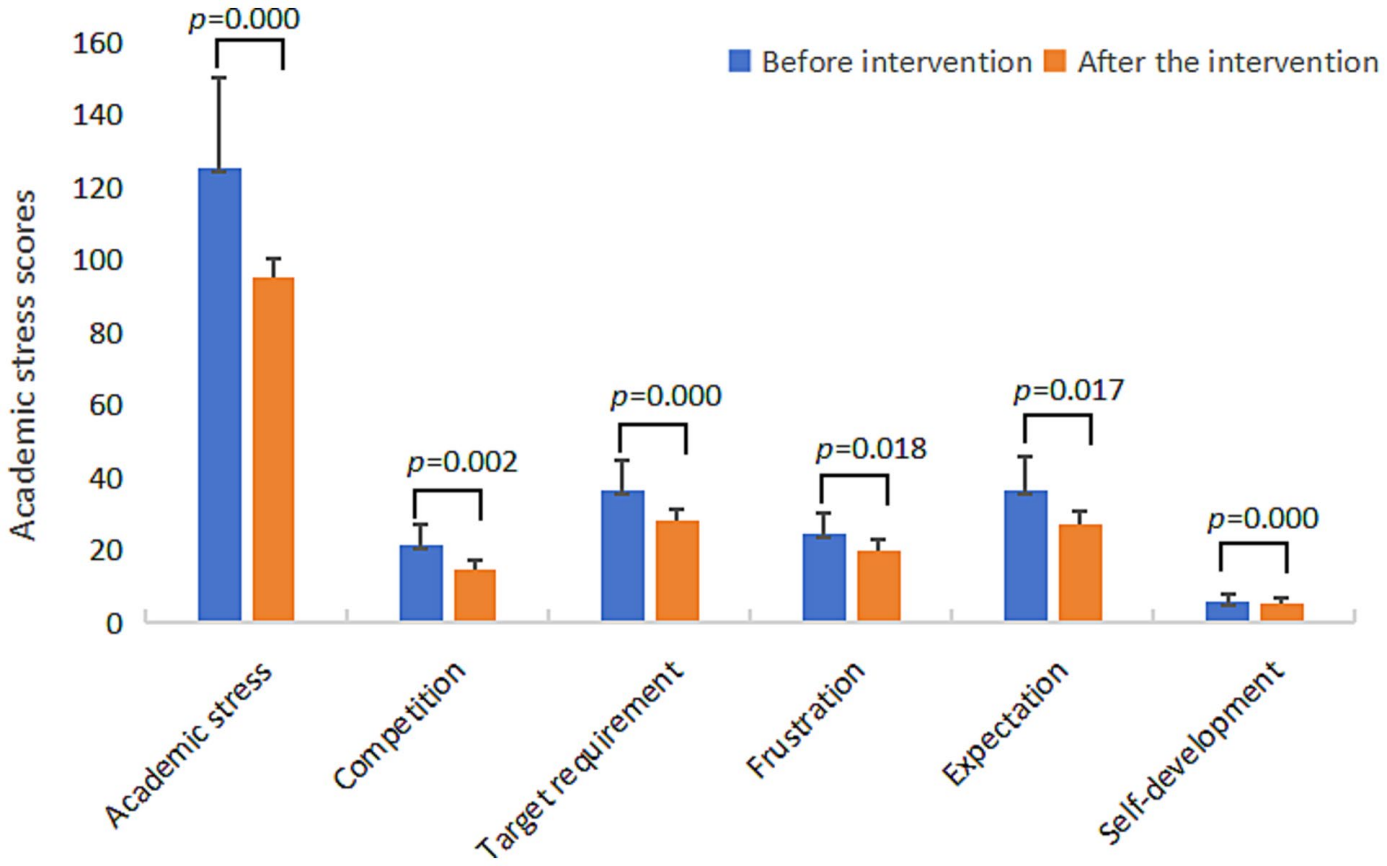
Bar chart of changes in academic stress scores of the experimental group before and after the intervention.

**Figure 4:**
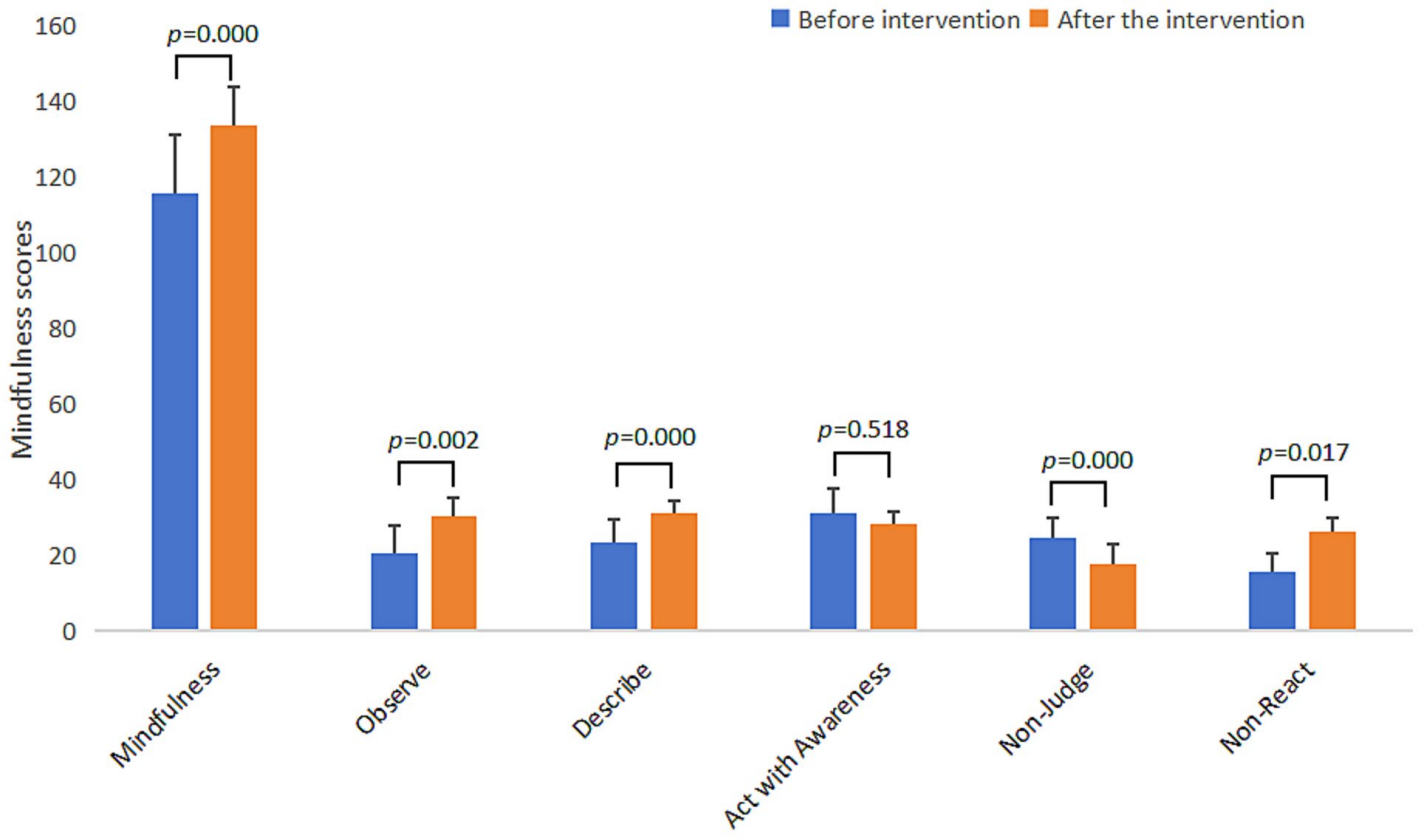
Bar chart of changes in mindfulness scores of the experimental group before and after the intervention.

Through the test of the difference between the experimental group and the control group (Table 4), it was found that there were significant differences between the experimental group and the control group in sleep quality, academic stress, and mindfulness, with statistically significant changes. Except for use of sleep medication (*t* = 1.52, *p* = 0.14) and act with awareness (*t* = 0.65, *p* = 0.52), the differences on other subscales were also significant. The changes in sleep quality, academic stress and mindfulness scores of the experimental group before and after the intervention are shown in Figures 2–4 respectively.

**Table 4:** Significance tests of experimental–control group contrasts of change scores (post–pre).

Variables		Change scores (post–pre)		*t*	*p*
		Experimental group	Control group		
Sleep quality		2.82 ± 1.74	0.13 ± 2.88	3.80	0.000***
	Subjective sleep quality	0.59 ± 0.96	0.04 ± 0.96	1.95	0.005**
	Sleep latency	0.64 ± 0.90	0.13 ± 1.08	1.74	0.009**
	Sleep duration	0.55 ± 0.60	−0.21 ± 0.88	3.36	0.002**
	Habitual sleep efficiency	0.23 ± 0.75	0.38 ± 0.50	3.24	0.002**
	Sleep disturbance	−0.36 ± 0.58	0.38 ± 0.77	−3.65	0.001**
	Use of sleep medication	0.18 ± 0.39	0.00 ± 0.42	1.52	0.137
	Daytime dysfunction	0.41 ± 0.80	−0.21 ± 0.93	2.41	0.020*
Academic stress		29.73 ± 1.38	25.09 ± 37.07	−4.31	0.000***
	Competition	6.50 ± 5.91	3.13 ± 7.73	−3.38	0.002**
	Target requirement	8.50 ± 8.35	−5.08 ± 13.06	−4.51	0.000***
	Frustration	4.91 ± 5.16	2.42 ± 12.05	0.65	0.018*
	Expectation	9.27 ± 10.24	0.63 ± 9.97	2.47	0.017*
	Self-development	0.55 ± 1.77	0.29 ± 3.41	−4.99	0.000***
Mindfulness		−18.00 ± 7.85	2.50 ± 14.35	−4.31	0.000***
	Observe	−9.68 ± 9.86	−0.92 ± 7.66	−3.38	0.002**
	Describe	−7.86 ± 6.97	1.21 ± 6.66	−4.51	0.000***
	Act with Awareness	3.05 ± 6.19	1.75 ± 7.19	0.65	0.518
	Non-Judge	−10.50 ± 6.02	−1.96 ± 5.58	−4.99	0.000***
	Non-React	7.00 ± 7.27	2.42 ± 5.22	2.47	0.017*

This table presents the results of independent-samples t-tests comparing the change scores (posttest minus pretest) between the experimental group and the control group across variables related to sleep quality, academic stress, and mindfulness. Values are presented as M ± SD. *p* < 0.05*, *p* < 0.01**, *p* < 0.001***.

## Discussion

The results of this study suggested that students who participated in the MBSR program showed significant improvements in their sleep quality, academic stress and mindfulness. Specifically, there was no significant difference in the variables between the experimental group and the control group before intervention. We compared sleep quality and academic stress of adolescents before and after intervention in the experimental group, which revealed a significant post-intervention improvement in the total score of these two variables. While there was no significant difference in before and after the intervention in control group. This shows that MBSR can improve sleep quality, relieve academic stress and achieve better expected results. This confirmed the hypothesis of this study and is consistent with the results of previous studies (Britton et al., 2012; Bei et al., 2013).

To verify that MBSR intervention is normative and effective, this study tested mindfulness scores before and after the intervention of all participants. The results show that 8-week of MBSR program could improve the mindfulness scores of the experimental group, and there was a significant difference in the scores before and after the intervention, while there was no significant change in the mindfulness scores of the control group. This suggested that the 8-week MBSR intervention process effectively improved the mindfulness level of the experimental group, which was consistent with the results of Kaplan et al. (2017), who believed that the effective mindfulness intervention was accompanied by the improvement of mindfulness level.

This study found the intervention effect of MBSR on sleep quality. Mindfulness intervention can improve self-reported subjective sleep quality, reduce the time to fall asleep, increase the subjective reported sleep time, and reduce sleep disorders and daytime dysfunction. Previous research has found that the cause of insomnia in adolescents in addition to puberty physiological factors, mainly poor mental health status, such as anxiety, depression, stress, etc., hypersomnia cognitive activities and bad sleep habits, such as playing video products, smoking, drinking tea, daytime sleepiness, etc. (Ma and Jiang, 2019). Mindfulness emphasizes that we pay attention to the present, focus on the objective feelings of the body, focus on the present moment, do not make value judgments on emotions and thoughts, trust ourselves, do not force ourselves, and accept the status quo (Yang et al., 2020). Anti-arousal mechanism can also partially explain this result (Spielman et al., 1987). Individuals with excessive arousal would increase the arousal degree of their central nervous system and increase the arousal time, so that they could not get a good sleep. Mindfulness intervention can change individuals’ attention strategies and degrees of attention to negative emotions, and change their response to emotions. The change of emotional attention, expression mode, action time and response can interfere with the cognitive process and behavioral pattern of insomnia, reduce the degree of sympathetic arousal, and thus achieve the effect of improving sleep quality (Jing et al., 2011). Research found the MBSR has no effect on use of sleep medication, the subjects in this study are adolescents, who may have some common sleep problems, but most of them do not have chronic sleep diseases and do not need hypnotic drugs to fall asleep. As a result, few people report the use of sleep medication. Moreover, the mechanism by which mindfulness improves sleep is mainly dependent on the effects of attention and cognition (Peng and Ju, 2013; Harvey, 2002), and neither attention nor cognition had a direct effect on the use of hypnotic drugs. Even for individual who reported, 8-week of MBSR showed no immediate or noticeable effect.

As expected, this study found that MBSR can reduce academic stress, adolescents who participated in MBSR program in competitive stress, target requirement stress, frustration stress, expectation stress and self-development stress have improved. The cognitive mechanism of mindfulness suggests that mindfulness intervention can change the cognitive pattern and cognitive degree. In the face of stress stimulation, individuals who have received formal and effective mindfulness training are more likely to adopt positive stress response strategies and view the origin of stress from a more objective perspective (Weinstein et al., 2009), thus reducing the negative effects of stress. Mindfulness emphasizes shifting attention from negative events and stress to oneself, so as to maintain a calm and stable state of mind, and show an attitude of acceptance to the stimuli around, and do not attach too much importance to a particular emotion or stimulus. When faced with stressful stimuli, individuals with high levels of mindfulness focus more on themselves and the present, and pay no attention to the negative effects of stress. In addition, this study found that after MBSR intervention, the level of mindfulness in the experimental group was also improved. Previous studies found that mindfulness can buffer stress and reduce negative evaluation of stress response (Creswell and Lindsay, 2014).

Considering the context of education in China, where students and parents place a greater emphasis on academic performance, there is often insufficient attention given to academic pressure and sleep quality. High societal expectations and a highly competitive environment may indirectly influence the effectiveness of interventions during the process. Therefore, when selecting participants for interventions, it is advisable to avoid graduating students as much as possible, and mindfulness-based stress reduction training should also be scheduled outside of examination periods.

Of course, there are several limitations in this study. This study relies entirely on participants’ subjective report. Although subjective report is a reasonable research method, future research should adopt more objective indicators of participants’ sleep and learning. Secondly, in the experimental design of this study, the control group did not complete any tasks. Although such design can effectively distinguish the two groups and reduce confusion, but the generalizability of the results is limited. Finally, the study also ignored the durability of MBSR interventions, the experimental group could be followed up in the future to further explore this issue.

Despite these limitations, the present study complements the empirical studies on MBSR by demonstrating the potential intervention effect of 8-week MBSR program on sleep quality and academic stress in adolescents. This study provides further evidence of the feasibility of the MBSR to help adolescents improve sleep quality and reduce academic stress in the school. In the intervention process, the participating students showed high enthusiasm and acceptance, which suggests that we can expand the application of MBSR in future mental health education. In addition to the sleep quality and academic stress involved in this study, it can also be applied to happiness, academic performance, emotional management, concentration and other aspects.

## Data Availability

The datasets presented in this study can be found in online repositories. The names of the repository/repositories and accession number(s) can be found in the article/supplementary material.
